# Age and 17β-Estradiol (E_2_) Facilitate Nuclear Export and Argonaute Loading of microRNAs in the Female Brain

**DOI:** 10.3390/ncrna9060074

**Published:** 2023-12-06

**Authors:** Megan L. Linscott, Yoldas Yildiz, Sarah Flury, Mikayla L. Newby, Toni R. Pak

**Affiliations:** Stritch School of Medicine, Loyola University Chicago, Maywood, IL 60153, USA; mlinscot@kent.edu (M.L.L.); yyildiz@luc.edu (Y.Y.); sflury@luc.edu (S.F.); mnewby1@luc.edu (M.L.N.)

**Keywords:** microRNA, RNA binding proteins, hnRNPA1, argonaute, estradiol, hypothalamus

## Abstract

Aging in women is accompanied by a dramatic change in circulating sex steroid hormones. Specifically, the primary circulating estrogen, 17β-estradiol (E_2_), is nearly undetectable in post-menopausal women. This decline is associated with a variety of cognitive and mood disorders, yet hormone replacement therapy is only effective within a narrow window of time surrounding the menopausal transition. Our previous work identified microRNAs as a potential molecular substrate underlying the change in E_2_ efficacy associated with menopause in advanced age. Specifically, we showed that E_2_ regulated a small subset of mature miRNAs in the aging female brain. In this study, we hypothesized that E_2_ regulates the stability of mature miRNAs by altering their subcellular localization and their association with argonaute proteins. We also tested the hypothesis that the RNA binding protein, hnRNP A1, was an important regulator of mature miR-9-5p expression in neuronal cells. Our results demonstrated that E_2_ treatment affected miRNA subcellular localization and its association with argonaute proteins differently, depending on the length of time following E_2_ deprivation (i.e., ovariectomy). We also provide strong evidence that hnRNP A1 regulates the transcription of pri-miR-9 and likely plays a posttranscriptional role in mature miR-9-5p turnover. Taken together, these data have important implications for considering the optimal timing for hormone replacement therapy, which might be less dependent on age and more related to how long treatment is delayed following menopause.

## 1. Introduction

Mammalian microRNAs (miRNAs) are small non-coding RNAs whose primary function in eukaryotes is to inhibit the translation of mRNA. In this way, miRNAs modulate most mammalian protein-coding mRNAs and, consequently, impact almost all essential cellular functions [1,2]. Canonically, primary miRNA transcripts (pri-miRNA) are cleaved by the RNase III enzyme DROSHA and then exported out of the nucleus, where they are further cleaved by a second RNase III enzyme called DICER. This process results in the formation of a ~22 nucleotide mature miRNA duplex that is loaded onto an argonaute (AGO) protein. AGO proteins cleave the duplex and then retain a single-strand miRNA to form the core of the miRNA-induced silencing complex (miRISC) [3,4]. The major catalytically active component of miRISC is AGO2; however, the mammalian argonaute family consists of four proteins (AGO1-4) all of which have the capacity to harbor miRNAs. Indeed, these alternative AGOs can bind terminal ends of small RNAs with their PAZ/MID domains, thereby making small RNAs inaccessible to endogenous nucleases and protecting them from degradation [5]. miRNAs are generally localized throughout the cytoplasm, although recent studies have demonstrated that some mature miRNAs, along with their bioprocessing components, are localized in a variety of specialized subcellular compartments, such as the nucleus, mitochondria, and stress granules. This shift in subcellular localization tends to be associated with cellular stress, senescence, and disease pathology. Importantly, the precise function of miRNAs in these subcellular compartments and their cellular trafficking mechanisms have not been fully elucidated. However, these data raise the possibility that sequestration in subcellular compartments helps to protect and stabilize the miRNA.

Notably, mammalian miRNA expression patterns are tissue-specific, change with age, and are influenced by a variety of cellular signaling molecules, all of which contribute to the breadth of their influence on fundamental physiological processes [2,6,7]. For instance, we previously demonstrated that the steroid hormone 17β-estradiol (E_2_) can post-transcriptionally regulate miRNAs and many of their predicted mRNA targets [6,8,9]. Specifically, we found that the ability of E_2_ to regulate miRNAs in the aged rat brain was dependent on the length of time between ovarian hormone depletion (via ovariectomy) and the subsequent timing of E_2_ replacement [6,9]. These observed miRNA expression patterns could be partly explained by E_2_-induced stability/degradation of select miRNAs, such as the neuronal-enriched miR-9-5p and miR-9-3p [8,10]. Moreover, we used mass spectrometry to reveal that mature miR-9-5p and miR-9-3p were bound to distinct groups of proteins, many of which were RNA binding proteins (RBP) [10], suggesting that the E_2_-mediated regulation of RBPs might explain how E_2_ stabilized only a select group of neuronal miRNAs. However, the role of RBPs and their function in miRNA regulation within the aging female brain remains unknown.

The primary goal of this study was to investigate possible mechanisms that explain how E_2_ regulates the expression of a small subset of miRNAs in the brain. To that end, we used a hypothalamic neuronal cell line and our established aged rat model of menopause to test the hypothesis that E_2_ induces differential loading of miR-9-5p onto the four AGO proteins and alters miR-9-5p subcellular localization following varying lengths of ovarian hormone deprivation. In addition, our previous work identified the RNA binding protein, hnRNP A1, as associating directly with miR-9-5p in neurons raising the possibility that hnRNP A1 mediates miR-9-5p stability. Therefore, we tested if miR-9-5p expression in hypothalamic neurons was dependent on hnRNP A1. Collectively, the data herein show that AGO loading of miR-9-5p is regulated by both E_2_ and the length of ovarian hormone deprivation, and that E_2_ altered miR-9-5p subcellular localization by facilitating nuclear export. Moreover, knockdown of hnRNP A1 significantly increased miR-9-5p expression, while simultaneously decreasing pri-miR-9, suggesting that hnRNP A1 might be important for regulating mature miR-9-5p turnover in neurons.

## 2. Results

### 2.1. The Length of Time Following Ovarian Hormone Deprivation Altered Which AGO Protein Was Most Associated with miR-9-5p in the Paraventricular Nucleus (PVN) of the Hypothalamus

**Figure 1:**
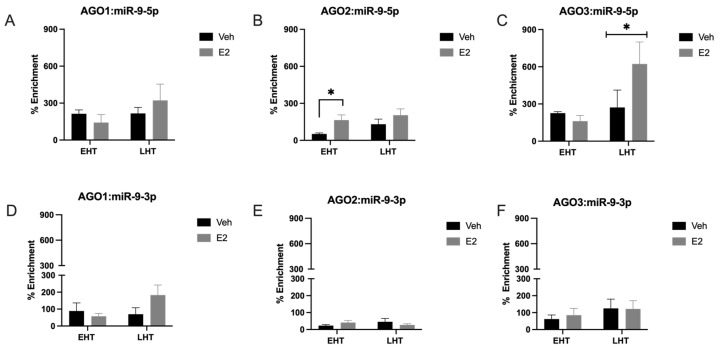
E_2_ shifted miR-9-5p association with argonaute proteins depending on length of ovarian hormone deprivation. The paraventricular nucleus of the hypothalamus was microdissected from aged rats treated with 17β-estradiol (E_2_) or safflower oil (Veh) at 1 week (early hormone treatment, EHT) or 4 weeks (late hormone treatment, LHT) post ovariectomy. Brain tissue samples were processed for RNA immunoprecipitation of miR-9-5p (**A**–**C**) or miR-9-3p (**D**–**F**) association with AGO1, AGO2, or AGO3. Data are depicted as mean ± SEM and analyzed by 2-factor ANOVA. An * indicates *p* < 0.05.

Mammals express four different AGO proteins, of which AGO2 is the most catalytically active, and recent data support the hypothesis that the different AGO proteins have unique cellular functions [11]. Therefore, we tested the hypothesis that E_2_ induces the differential association of miR-9-5p and miR-9-3p with AGO1, 2, 3, and 4 in the brain. To test this hypothesis, we used RNA Immunoprecipitation (RIP) assays to quantify the association of miR-9-5p and miR-9-3p with AGO1, AGO2, AGO3, and AGO4 in our aged rat model of menopause with early (EHT) or late (LHT) hormone replacement therapy following ovariectomy (OVX). The association of miR-9-5p and miR-9-3p with each AGO protein was quantified using whole cell lysate dissected from the paraventricular nucleus (PVN) of the hypothalamus. Our results demonstrated that both miR-9-5p and miR-9-3p were associated with AGO1, AGO2, and AGO3, but not with AGO4 (Figure 1; AGO4 not shown). In fact, AGO4 was undetectable in both vehicle and E_2_-treated rats, which is consistent with other studies showing little to no expression of AGO4 in all central nervous system (CNS) cell types [12]. Overall, AGO2 had the lowest percent enrichment of miR-9-5p and miR-9-3p compared to AGO1 and AGO3 (Figure 1A–C), perhaps indicating different kinetics of miRNA turnover between the different AGO proteins. Treatment with E_2_ significantly increased miR-9-5p association with AGO2 in animals treated early after OVX (EHT), but not in animals treated late (LHT, i.e., prolonged E_2_ deprivation (Figure 1B). Moreover, E_2_ treatment significantly increased miR-9-5p association with AGO3 in the LHT group, but not in the EHT group (Figure 1C). There were no effects of E_2_ treatment in miR-9-5p association with AGO1, or in miR-9-3p association with AGO1, AGO2, or AGO3 in either group (Figure 1D–F). Overall, our results demonstrated that miR-9-5p was preferentially loaded onto AGO3 (Figure 1C). For example, miR-9-5p was loaded onto AGO3 at almost double the capacity of AGO2, while AGO2 enrichment was lowest overall.

### 2.2. The Length of Time Following Ovarian Hormone Deprivation Altered Subcellular miR Localization in PVN-Derived Neuronal Cells

Recent studies have shown that miRNAs shuttle between a variety of subcellular compartments in response to the activation of signaling pathways that impact homeostasis, such as cellular stress. Our previous work demonstrated that E_2_ affected the stability and expression of a small subset of miRNAs dependent on age and length of ovarian hormone deprivation, and aging is a well described cellular stressor. Therefore, we hypothesized that E_2_ would alter miR-9-5p and miR-9-3p subcellular localization dependent on the length of time following ovarian hormone depletion via OVX in aged rats.

**Figure 2:**
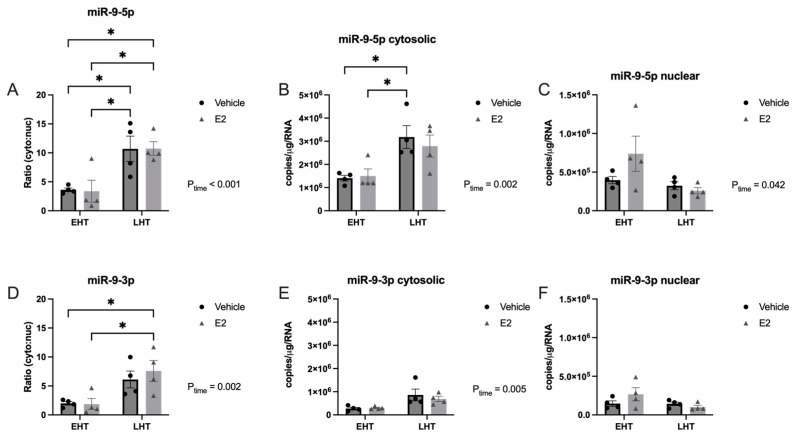
Subcellular localization of miR-9-5p and miR-9-3p was altered following longer periods of ovarian hormone deprivation and E_2_ treatment. The paraventricular nucleus of the hypothalamus was microdissected from aged rats treated with 17β-estradiol (E_2_) or safflower oil (Veh) at 1 week (early hormone treatment, EHT) or 4 weeks (late hormone treatment, LHT) post ovariectomy. Tissue samples were freshly dissected and separated into nuclear and cytosolic fractions. Fractions were processed for RT-qPCR and absolute levels quantified based on extrapolation from a known standard curve for miR-9-5p (**A**–**C**) and miR-9-3p (**D**–**F**). Data are depicted as mean ± SEM and analyzed by 2-factor ANOVA. An * indicates *p* < 0.05.

To test this hypothesis, we first quantified the absolute amount of mature miR-9-5p and miR-9-3p in the nucleus and cytoplasm of brain tissue freshly isolated from the PVN in our aged rat model of menopause. Our results demonstrated that the length of time following E_2_ deprivation significantly increased the absolute amount of miR-9-5p in the cytoplasm (Figure 2A,B), which was also reflected by a significant decrease in the amount of nuclear miR-9-5p (Figure 2A,C).

This subcellular shift was further supported by a statistically significant increase in the cytosolic:nuclear ratio observed in the LHT group (Figure 2A). However, E_2_ treatment had no additional effect on the amount of miR-9-5p in either subcellular compartment regardless of the length of time post-OVX (Figure 2A–C). Our data also revealed a statistically significant main effect of time on E_2_ deprivation in the amount of cytosolic miR-9-3p, but not in the nucleus (Figure 2D–F), which was verified by a significant increase in the cytosolic:nuclear ratio in the LHT group (Figure 2D).

**Figure 3:**
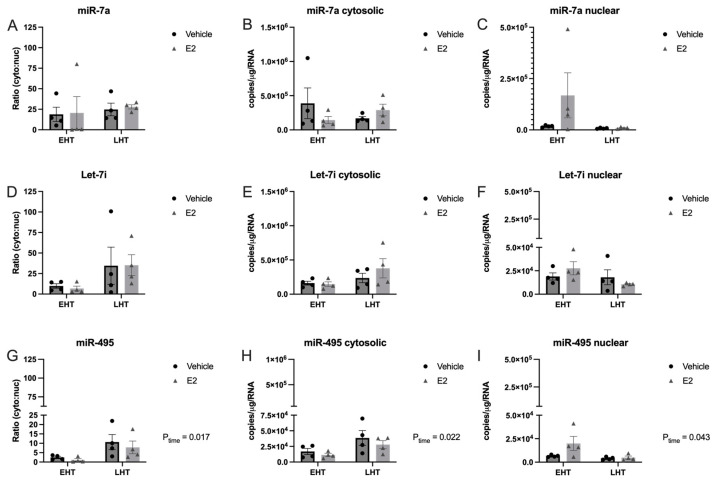
Subcellular localization of miR-495 was altered following longer periods of ovarian hormone deprivation. The paraventricular nucleus of the hypothalamus was microdissected from aged rats treated with 17β-estradiol (E_2_) or safflower oil (Veh) at 1 week (early hormone treatment, EHT) or 4 weeks (late hormone treatment, LHT) post ovariectomy. Tissue samples were freshly dissected and separated into nuclear and cytosolic fractions. Fractions were processed for RT-qPCR and absolute levels quantified based on extrapolation from a known standard curve for miR-7a (**A**–**C**), let-7i (**D**–**F**) and mir-495 (**G**–**I**). Data are depicted as mean ± SEM and analyzed by 2-factor ANOVA. There was a statistically significant main effect of time for miR-495.

Next, we quantified the subcellular distribution of three additional miRNAs that we had previously shown were regulated by E_2_ in an age-dependent manner: miR-7a, let-7i, and miR-495 (Figure 3).

Notably, only miR-495 showed a statistically significant subcellular shift from the nucleus to the cytoplasm that was dependent on the length of time post-OVX (Figure 3G–I). The results of miR-495 were consistent with miR-9-5p and miR-9-3p such that longer periods of E_2_ deprivation induced a statistically significant shift of miRNAs out of the nucleus, but the shift was not altered following the subsequent E_2_ treatment. Moreover, the miR-495 mean subcellular cytosolic:nuclear ratio in the EHT group showed that it was more equally distributed between the two subcellular compartments compared to miR-7a and let-7i (Figure 3). It was also evident that miR-495 was expressed at an order of magnitude lower than all the other miRNAs measured in the PVN, as denoted with a split Y-axis (Figure 3G–I).

### 2.3. E_2_ Treatment Altered the Subcellular Localization of let-7i in a Hypothalamic Neuronal Cell Line Derived from the Paraventricular Nucleus (PVN) of the Hypothalamus

**Figure 4:**
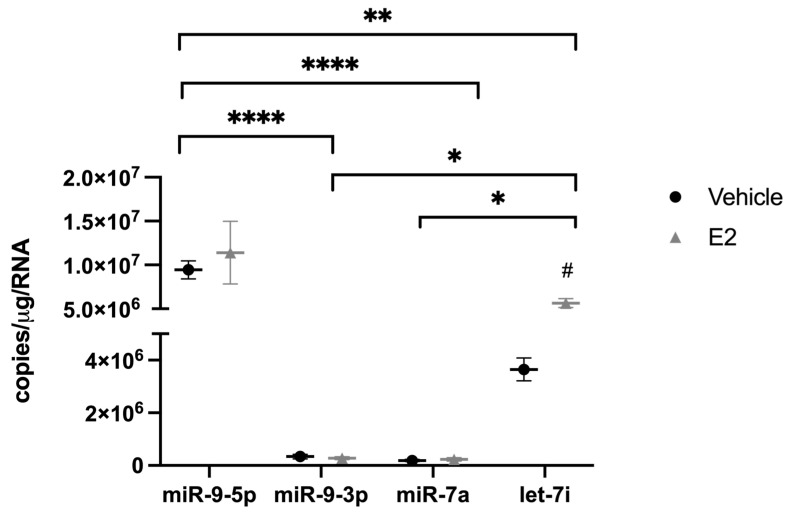
Comparison of absolute expression levels for E_2_-regulated miRNAs in the hypothalamic-derived neuronal cell line, IVB. Absolute levels of miR-9-5p, miR-9-3p, miR-7a, and let-7i were quantified by RT-qPCR from cells treated with E_2_ for 16 h prior to lysis and RNA isolation. Data are depicted as mean ± SEM and analyzed by one-factor ANOVA. * *p* < 0.05; ** *p* < 0.01; **** *p* < 0.0001.

Expression of mature miR-9-5p/3p is nearly exclusively found in neurons and our PVN tissue contained a mixture of glia and neuronal cell types. Therefore, we used a neuronal cell line derived from the PVN (IVB cells) to specifically isolate the effects of E_2_ on miRNA subcellular localization in neurons. The IVB cells were grown in charcoal-stripped fetal bovine serum (FBS) for 48 h to remove the steroid hormones present in normal FBS. Cells were then treated with E_2_ or vehicle for 16 h prior to cellular fractionation of the nucleus and cytosol. Fraction purity was verified by qPCR for ribosomal RNA 18S and snoRNA U2 for cytoplasmic and nuclear compartments, respectively. First, we measured the absolute levels of miR-9-5p, miR-9-3p, miR-7a, let-7i, and miR-495 in the IVB cell line (Figure 4).

**Figure 5:**
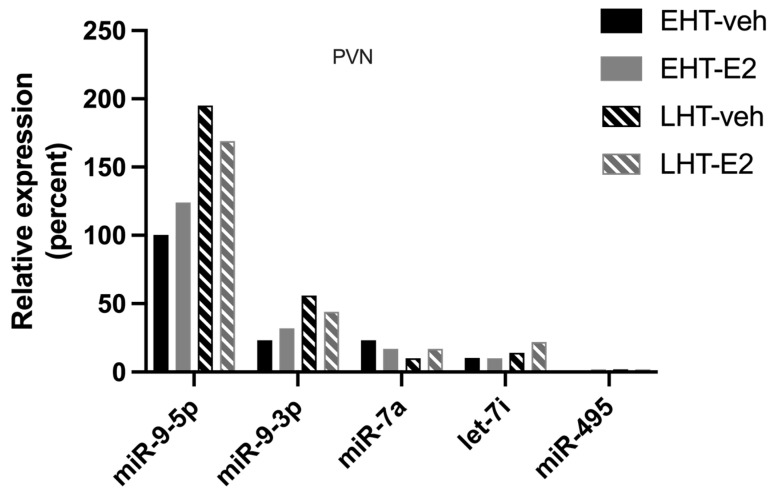
Comparison of relative expression levels for E_2_-regulated miRNAs in the paraventricular nucleus of the hypothalamus in aged female rats. The paraventricular nucleus of the hypothalamus was microdissected from aged rats treated with 17β-estradiol (E_2_) or safflower oil (Veh) at 1 week (early hormone treatment, EHT) or 4 weeks (late hormone treatment, LHT) post ovariectomy. Relative levels of miR-9-5p, miR-9-3p, miR-7a, let-7i, and miR-495 were quantified by RT-qPCR. Data are depicted as percent difference compared to miR-9-5p (early vehicle treatment).

Our data showed that miR-9-5p was significantly more abundant than all other miRs tested, which was consistent with published studies showing that miR-9 is enriched in neurons [13]. The next most abundant was let-7i, followed by miR-9-3p and miR-7a at equivalently low levels, and there were no detectable levels of miR-495 (Figure 4, miR-495 not shown). These data were consistent with the relative abundance of each of these miRNAs that we observed in the PVN tissue isolated from our rat model of menopause (Figure 5).

**Figure 6:**
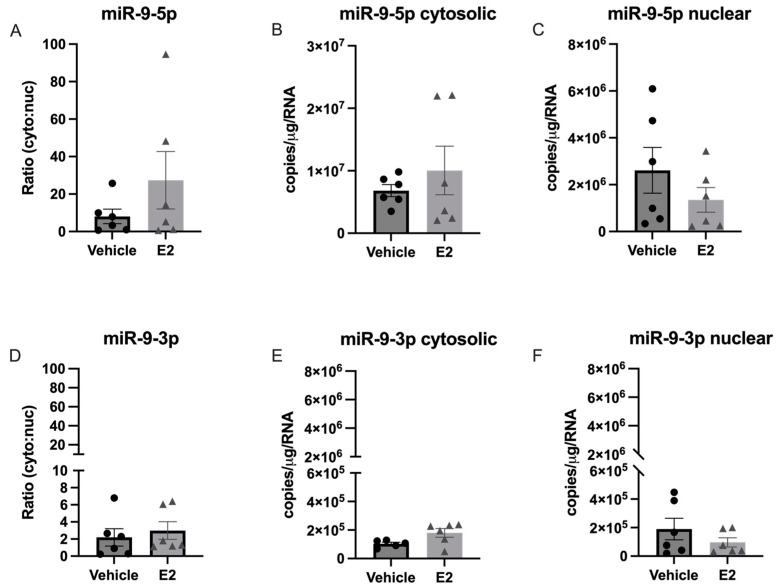
E_2_ treatment did not alter subcellular localization of miR-9-5p and miR-9-3p in hypothalamic-derived IVB cells. Cells were treated with E_2_ for 16 h prior to lysis, cellular fractionation, and RNA isolation. Fractions were processed for RT-qPCR and absolute levels of miR-9-5p (**A**–**C**), and miR-9-3p (**D**–**F**) were quantified based on extrapolation from a known standard curve. Data are depicted as mean ± SEM and analyzed by one-factor ANOVA.

Next, we quantified nuclear and cytosolic levels of each miR and tested if E_2_ treatment altered the cytosolic:nuclear ratio. In contrast to what we observed in our aged rat model of menopause, there was no significant change in the cytosolic:nuclear ratio of miR-9-5p or miR-9-3p in IVB cells treated with E_2_ (Figure 6).

**Figure 7:**
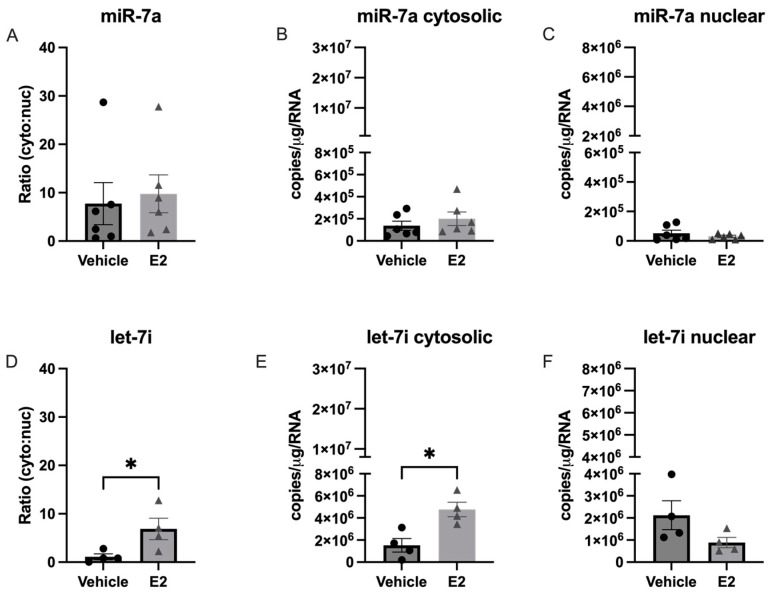
E_2_ treatment altered subcellular localization of let-7i, but not miR-7a in hypothalamic-derived IVB cells. Cells were treated with E_2_ for 16 h prior to lysis, cellular fractionation, and RNA isolation. Fractions were processed for RT-qPCR and absolute levels of miR-7a (**A**–**C**), and let-7i (**D**–**F**) were quantified based on extrapolation from a known standard curve. Data are depicted as mean ± SEM and analyzed by one-factor ANOVA. An * indicates *p* < 0.05.

We then quantified the cytosolic:nuclear ratio of miR-7a and let-7i in the IVB cells (Figure 7). Our results showed that E_2_ treatment induced a significant shift in let-7i, but not miR-7a, from the nucleus to the cytoplasm (Figure 7E), which was not observed in our animal model, suggesting that the effects of E_2_ in hypothalamic neurons could be masked by other cell types present in the hypothalamus. Finally, our animal model showed that the length of time following ovarian hormone deprivation significantly altered the cytosolic:nuclear ratio for miR-495, but this miR was not expressed at high enough levels in the IVB cell line to determine if that effect was specific to neurons.

### 2.4. E_2_ Treatment Decreased miRNA Association with AGO1 and AGO2 in the Nucleus in PVN-Derived Neuronal Cells

**Figure 8:**
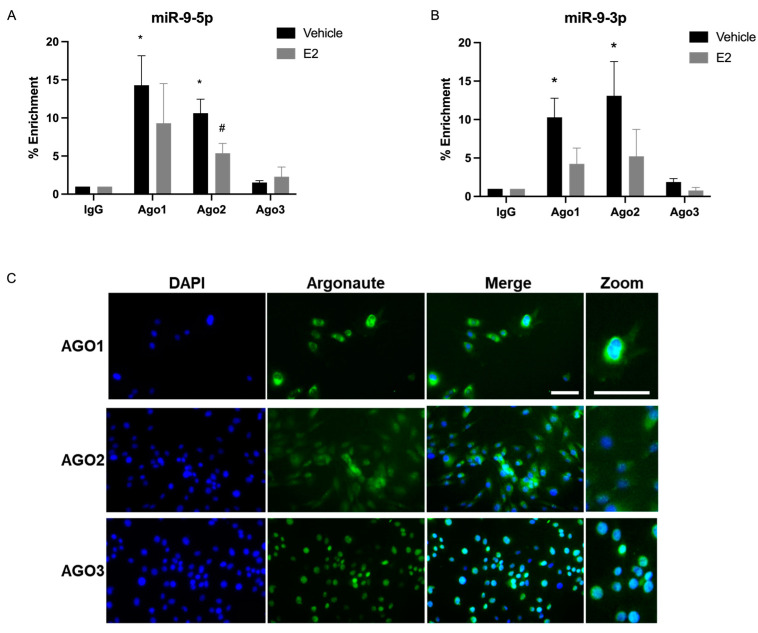
E_2_ shifted miR-9-5p and miR-9-3p association with argonaute proteins in the nucleus of hypothalamic-derived IVB cells. Nuclear fractions of cell lysates were processed for RNA immunoprecipitation of miR-9-5p (**A**) or miR-9-3p (**B**) association with AGO1, AGO2, or AGO3. Data are depicted as mean ± SEM and analyzed by two-factor ANOVA. An * indicates *p* < 0.05 between groups; # indicates *p* < 0.05 within groups. (**C**) Immunofluorescence for AGO1, AGO2, and AGO3 in IVB cells. Green = AGO proteins; blue = DAPI. Scale bar = 50µm.

**Figure A1:**
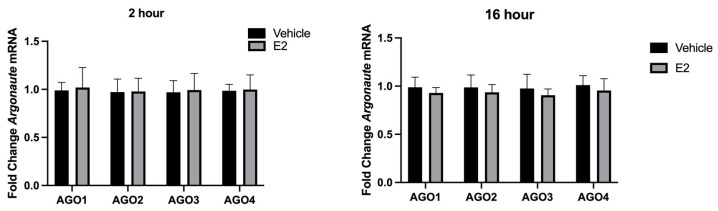
E_2_ treatment did not alter expression of AGO. Relative expression of AGO1, AGO2, AGO3, and AGO4 were quantified by RT-qPCR in hypothalamic IVB cells treated with vehicle or 10 nM E_2_ for 2 (left panel) or 16 (right panel) hours (N = 3 biological and 6 technical replicates/treatment/time). Data are depicted as mean ± SEM.

The results we observed in the IVB cells and in the hypothalamic PVN of our animal model demonstrated that there was a relatively high abundance of miR-9-5p and miR-9-3p in the nucleus (Figure 2). Moreover, there is evidence that argonaute proteins are present in the nucleus of many cell types, although the full scope of nuclear AGO functions remain unclear [14,15,16,17,18]. To determine if these miRNAs were differentially associated with nuclear AGO1, 2, or 3, we quantified the percent enrichment of miR-9-5p/3p associated with these AGOs in the nucleus (Figure 8). We also tested if E_2_ treatment would dictate a preferential association for a specific AGO protein in the nucleus. Consistent with our previous studies, AGO expression was not affected by E_2_ treatment (Figure A1). The data showed that miR-9-5p and miR-9-3p were equally enriched with AGO1 and AGO2, both in the presence and absence of E_2_, and there was no enrichment with nuclear AGO3 (Figure 8A,B). This was in sharp contrast to our animal model where we observed the highest association of miR-9-5p with AGO3 in whole tissue PVN lysate (Figure 1C). Moreover, E_2_ treatment decreased the association of miR-9-5p, but not -3p, with nuclear AGO2 (Figure 8A).

We next used immunofluorescence to determine if the lack of miR-9-5p/3p association with AGO3 in the nucleus was due a lack of nuclear AGO3 expression in the nucleus. Figure 8C shows that AGO1 and AGO2 had modest nuclear and high perinuclear localization (DAPI nuclear = blue; argonaute = green). By contrast, AGO3 appears to have a very high expression in the nucleus based on the merged image. Together, these results suggest that miR9-5p and miR-9-3p preferentially associate with cytoplasmic, and not nuclear, AGO3 and that this is not due to the lack of AGO3 availability in the nucleus.

### 2.5. hnRNP A1 Decreased Mature miR-9-5p Expression in PVN-Derived Neuronal Cells

Our previous work showed that miR-9-5p and miR-9-3p were regulated by E_2_ in an age-dependent manner [6,9], and that miR-9-5p was overall less stable than miR-9-3p [10]. Further, we used a proximity-dependent biotinylation approach combined with mass spectrometry to identify RNA binding proteins associated with miR-9-5p and miR-9-3p to determine possible mechanisms for the observed differences in stability of miR-9-5p/3p in neurons [8]. One of the main RBPs associated with miR-9-5p, but not miR-9-3p, was hnRNP A1, and we hypothesized that hnRNP A1 binding helped to stabilize mature miR-9-5p in the cytoplasm. To test this hypothesis, we used siRNA to knockdown hnRNP A1 in IVB cells and then measured miR-9-5p expression with RT-qPCR.

**Figure 9:**
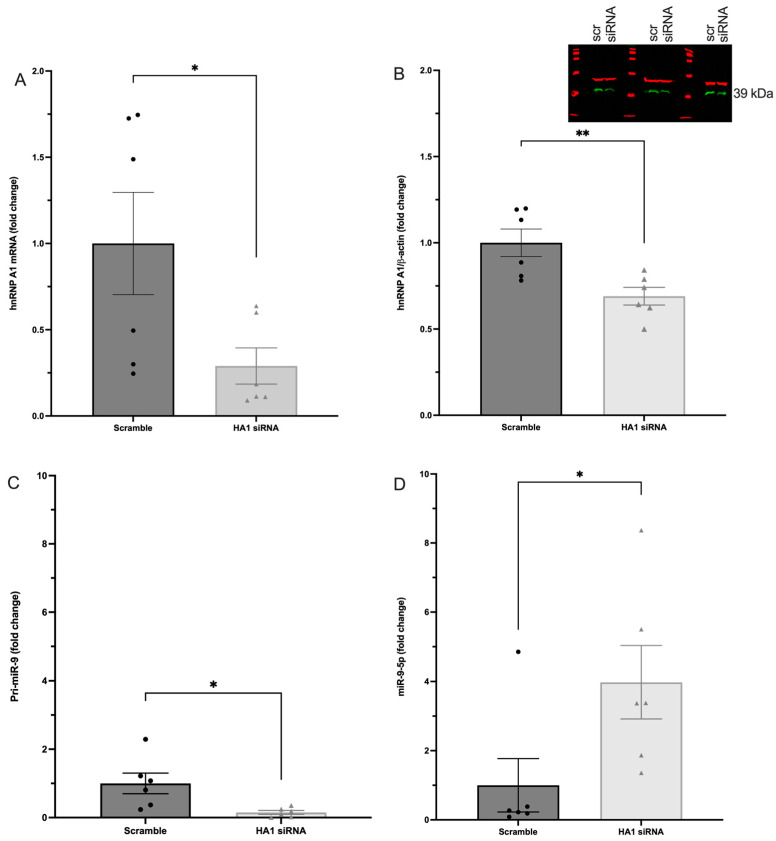
hnRNPA1 knockdown reduced pri-miR-9 and increased mature miR-9-5p expression in hypothalamic-derived IVB cells. (**A**) RT-qPCR and (**B**) Western blot analysis of hnRNP A1 expression in cells 48 h after transient transfection with hnRNP A1 siRNA (HA1) or scramble (control). (**C**) RT-qPCR analysis for pri-miR-9 and (**D**) mature miR-9-5p in cells 48 h after transient transfection with hnRNP A1 siRNA (HA1) or scramble (control). Data are depicted as fold change compared to scramble siRNA treatment and analyzed using Student’s *t*-test. * Indicates *p* < 0.05. ** indicates *p* < 0.01.

First, we showed that siRNA against hnRNP A1 resulted in a ~75% and ~30% decrease in mRNA and protein levels, respectively (Figure 9A–C). We then measured the effects of hnRNP A1 knockdown on pri-miR-9 and mature miR-9-5p levels. The data showed a statistically significant reduction in pri-miR-9 following hnRNP A1 knockdown (Figure 9D), which is consistent with the reported effects of hnRNP A1 on miRNA processing [19,20,21]. However, contrary to our hypothesis, we observed an unexpected increase in mature miR-9-5p expression following hnRNP A1 knockdown (Figure 9D), suggesting that hnRNP A1 might destabilize miR-9-5p in the cytoplasm. These results could explain our previous observations that miR-9-5p was inherently less stable than miR-9-3p in neuronal cells.

## 3. Discussion

### 3.1. Summary of Major Findings

The goal of these studies was to further our understanding of how E_2_ post-transcriptionally regulates the expression of a small subset of mature miRNAs in the aging female brain. Our data revealed three major novel findings. First, we demonstrated that E_2_-regulated miRNAs are differentially associated with diverse argonaute proteins in the aged brain, and these associations are dependent on the length of time following ovarian hormone deprivation. Unexpectedly, AGO3, and not AGO2, was the primary argonaute protein associated with miR-9-5p in the aged brain. Moreover, the association with AGO3 was restricted to cytoplasmic AGO3, whereas nuclear miR-9-5p/3p were associated only with AGO1 and AGO2. Collectively, our results suggest that different argonaute proteins preferentially load specific miRNAs depending on the miRNA subcellular localization, which is regulated, in part, by age and hormonal milieu. Second, our results showed that long periods of ovarian hormone deprivation caused a significant shift of miRNAs out of the nucleus, which would predict a substantive impact on their biological function. One possible mechanism driving miRNA shuttling out of the nucleus is through its association with RNA binding proteins (RBP)s, several of which we recently identified as associating with miR-9-5p [8]. The data herein provide evidence that one of these RBPs, hnRNP A1, might facilitate miR-9-5p biogenesis, nuclear export, and turnover, because knocking down hnRNP A1 led to decreased pri-miR-9 and increased mature miR-9-5p. Collectively, these data are the first to describe the impact of age and E_2_ on AGO association and subcellular localization for these miRNAs, and revealed that hnRNP A1 regulates both the pri- and mature miR-9-5p.

### 3.2. Length of Ovarian Hormone Deprivation Regulates mi-9-5p AGO Switching in the Aged Female Brain

Our results showed that miR-9-5p is preferentially loaded onto AGO3, and not the predicted AGO2, with advanced age and after long periods of E_2_ deprivation in the female brain. Consistent with our findings, studies in drosophila demonstrated that aging alone caused a shift in miRNA loading from the preferred AGO1 to AGO2 [22]. One possible explanation is that specific miRNAs can establish a preference for an AGO interaction that is mediated by their end terminal modifications [22], and it is not unreasonable to predict that these modifications could be altered by age and/or hormonal milieu [23]. Unexpectedly, miR-9-5p was loaded onto AGO3 at almost double the capacity of AGO2, while the AGO2:miR-9-5p enrichment was lowest overall. Until recently, AGO2 was thought to be the only argonaute possessing mRNA slicer activity; however, AGO3 has now been reported to have mRNA slicer activity but with specific substrate requirements, specifically on the post-seed sequence [24]. The shift in mi-9-5p preference for AGO3 after long periods of E_2_ deprivation in advanced age could result in decreased binding to canonical miR-9-5p mRNA targets, hence the aberrant overexpression of those genes, and perhaps a bias towards binding alternative mRNA targets, resulting in inappropriate gene silencing. Furthermore, the overall lowered magnitude of enrichment with AGO2 suggests that miR-9-5p is rapidly chaperoned to its target mRNA and then dissociated from AGO2, whereas the association with AGO3 is more stable. E_2_ treatment also increased miR-9-5p loading onto AGO2; however, increasing the length of hormone deprivation had no significant effect. This observation may account for the differences in mRNA and protein expression that we previously observed in our ovarian hormone deprivation model. Overall, the physiological consequences of differential AGO loading in advanced age could contribute to age-related neurobiological diseases through dysregulation of normal miRNA silencing mechanisms.

Contrary to the results observed for miR-9-5p, neither age nor E_2_ treatment altered AGO1, AGO2, or AGO3 association with its antisense “passenger” strand, miR-9-3p, indicating that miR:AGO preference is miRNA-specific, and “AGO switching” may require unique *cis* and/or *trans* factors. There is some evidence that AGO3 is biased towards loading the antisense strand of certain miRNAs [25], but this was not observed for miR-9-3p, either in our animal model or in the neuronal cell line. Rather, our results are consistent with studies showing that a subset of miRNAs display an AGO loading bias that is likely dependent on *cis*-sequence differences at either the 5′ or 3′ end [26,27,28]. For example, miR-222 has a longer variant with four extra nucleotides at the 3′ end. This variant is more highly associated with AGO1 compared to the shorter miR-222 isomiR that associates equivalently with AGO2 and AGO3 [27]. Similarly, some mature forms of miR-9-5p also have altered end-terminal sequences. Specifically, pri-miR-9 can be transcribed from locations on three separate chromosomes creating miR-9-1, miR-9-2, and miR-9-3 paralogs [13]. A recent study demonstrated that miR-9-1 is differentially cleaved by Drosha resulting in a miR-9-5p isoform with a slightly shifted seed sequence [29]. Although our current RIP results could not discern from which chromosomal loci our mature miR-9-5p was derived, our prior studies showed that pri-miR-9-1 and pri-miR-9-2 were uniquely regulated by age and the length of E_2_ deprivation [9]. Taken together, these data raise the possibility that an isomiR derived from the E_2_-regulated miR-9-1 paralog explains our observed AGO loading bias for miR-9-5p.

### 3.3. miR-9-5p and miR-9-3p Preferentially Associate with Cytoplasmic, and Not Nuclear, AGO3 in a Hypothalamic-Derived Neuronal Cell Line

One of our most notable observations was that miR-9-5p and miR-9-3p did not associate with nuclear AGO3, despite apparent high levels of AGO3 nuclear expression in the hypothalamic PVN-derived IVB neuronal cells. This result was in stark contrast to the relatively high enrichment we observed for miR-9-5p:AGO3 in vivo using whole PVN tissue. AGO3 is best studied for its role in piRNA biogenesis as a key component of the ping-pong cycle in germ cells [30,31]. However, in that context, AGO3 is associated in the perinuclear region within a membraneless cytoplasmic organelle called nuage, and there is no evidence of intranuclear AGO3 expression in germ cells [30]. Conversely, AGO3 was consistently found to be associated with AGO2 in the nucleus of the human breast cancer cell line T47D [32]. These data demonstrate that there are likely cell type and/or developmental stage-specific subcellular localization of AGO proteins dependent on their respective biological functions. Moreover, the complete lack of miR-9-5p/3p loading onto nuclear AGO3 raises the intriguing possibility that miRNA loading mechanisms are different in the cytoplasm compared to the nucleus.

### 3.4. Length of Ovarian Hormone Deprivation Alters miRNA Subcellular Localization in the Aged Female Brain

The detection of miR-21 in the nucleus of HeLa cells was the first evidence to suggest that there could be a biological function for miRNAs localized to cellular regions outside of the cytoplasm [33]. Subsequent studies have since showed that miRNAs are widely distributed throughout the cell in multiple subcellular compartments, and that relocation can be induced by cellular stress, disease state, and neuronal activity [34,35]. However, to our knowledge there have been no prior reports demonstrating that age and/or steroid hormones can induce changes in miRNA subcellular localization. We previously showed that E_2_ shifted miR-9-5p and miR-9-3p polysome occupancy in vitro, which logically predicted that E_2_ could induce differential subcellular localization. The experiments herein using our aged female rat model of menopause confirmed that the length of time following ovarian hormone deprivation was the driving factor for alterations in the cytoplasmic:nuclear localization of miR-9-5p and miR-9-3p in the hypothalamus. Moreover, the effect of ovarian hormone deprivation on miRNA subcellular localization was specific to miR-9-5p/3p and not for other miRNAs previously reported to be E_2_ regulated, suggesting that other E_2_-regulated cellular components are important for conferring miRNA specificity. The biological consequences of miRNA cellular relocalization post-menopause are unclear; however, the nuclear functions of miRNAs are notably different from their canonical mRNA silencing role in the cytoplasm. For instance, nuclear miRNAs can bind directly to gene promoters or tether as a trans-acting factor to enhance and/or repress gene transcription [34,36,37,38,39,40]. Therefore, actively shuttling miRNAs to the cytoplasm or the nucleus could increase the biological function in one compartment at the expense of reducing the other (i.e., increased/decreased mRNA silencing).

### 3.5. The RNA Binding Protein, hnRNP A1, Is an Important Regulator of miR-9 Expression in Hypothalamic PVN-Derived Neurons

The hnRNP family of RNA binding proteins are key multifunctional proteins essential for all aspects of RNA metabolism including RNA splicing, RNA trafficking, and miRNA biogenesis. Moreover, hnRNP A1 is highly abundant in neuronal cells and its dysregulation has been implicated in several neurodegenerative diseases [41,42]. Our previous data showed that miR-9-5p binds hnRNP A1, likely through one of the two RNA-binding domains on the hnRNP A1 protein. Here, we tested the functional implications of hnRNP A1 association with miR-9-5p by knocking down levels of hnRNP A1 using siRNA in our hypothalamic-derived neuronal cell line. Here, we demonstrated that a reduction in hnRNP A1 caused a concomitant decrease in pri-miR-9 expression, suggesting that hnRNP A1 plays a role in the transcriptional regulation of miR-9. These data are consistent with hnRNP A1’s previously described role in modulating gene transcription, including genes that encode miRNAs [43,44,45,46]. Other studies have shown that hnRNP A1 is a positive regulator of miRNAs. Specifically, it binds to the terminal loop of select primary miRNA transcripts, such as miR-18a, and enhances the efficiency of the Drosha cleavage to increase miR-18a levels [47,48]. By contrast, our data herein raise the novel possibility that hnRNP A1 has a negative regulatory function for miR-9 in neurons because siRNA knockdown of hnRNP A1 significantly increased the levels of miR-9-5p. These data point to hnRNP A1 as a potential factor in regulating mature miR-9-5p degradation in the cytoplasm, which is consistent with our previous mass spectrometry data demonstrating that hnRNP A1 interacts with mature miR-9-5p [10].

## 4. Materials and Methods

### 4.1. Animals

Animal procedures were approved by the Institutional Animal Care and Use Committee (IACUC) at Loyola University Chicago (#2009018). All necessary measures were taken to minimize the pain and suffering of animals subject to the experimental procedures. Eighteen-month-old Fischer 344 rats were obtained from the National Institutes of Aging (NIA) colony at Charles River Laboratories. Rats were pair-housed upon arrival and allowed to acclimate to their environment for one week prior to further experimentation. Rats were supplied with standard rat chow and tap water ad libitum and were kept on a 12/12 h light/dark cycle with Zeitgeber time (ZT) 0 at 7 A.M. Animals were ovariectomized (OVX) at 18 months of age after the acclimation period and then left undisturbed for 1 (early hormone treatment, EHT group) or 4 weeks (late hormone treatment, LHT group) following OVX. After the designated time interval post-OVX, the animals were given a subcutaneous injection of either safflower oil or 2.5 mg/kg 17β-estradiol (E_2_) dissolved in safflower oil once/day for 3 consecutive days. This dose has been previously reported to achieve circulating E_2_ concentrations within the physiological range for postmenopausal women receiving HRT (17–75 pg/mL) [9,49,50]). Animals were euthanized 24 h after the final injection.

#### 4.1.1. Ovariectomy

Animals were deeply anesthetized with vaporized isoflurane and bilaterally ovariectomized (OVX) as described previously [9]. Briefly, the ovary and distal end of the uterine horn were excised from the body cavity after the uterine horn was clamped with a hemostat and ligated proximal to the clamp. Animals were singly housed and provided with acetaminophen analgesic in tap water for 3 days following the procedure. After 3 days of analgesia, the animals were pair-housed with their previous cage mate. Surgical wound clips were removed 10 days post-OVX under brief, light isoflurane anesthesia. Animals were then undisturbed for the duration of the experiment.

#### 4.1.2. Tissue Processing

The animals were deeply anesthetized using vaporized isoflurane and euthanized by rapid decapitation. Brains were rapidly dissected, flash-frozen in 2-methylbutane at −30 °C, and then sectioned coronally at 200 μm on a freezing microtome (Leica Biosystems, Lincolnshire, IL, USA). The paraventricular nucleus (PVN) was microdissected using a 0.75 mm Palkovit’s brain punch tool (Stoelting, Inc., Wood Dale, IL, USA) at −1.49 to −2.12 relative to bregma, as defined by The Rat Brain in Stereotaxic Coordinates [51]. Frozen PVN microdissections were transferred to a microcentrifuge tube and stored at −80 °C.

### 4.2. Cell Culture

For in vitro studies, we used a neuronal cell line derived from the paraventricular nucleus (PVN) of the rat hypothalamus (IVB cells, originally provided by John Kaskow, University of Cincinnati, OH, USA). Cells were maintained in normal growth media (DMEM media containing glucose, L-glutamine, sodium pyruvate, and 10% fetal bovine serum (FBS)) and grown to 60–70% confluency prior to experiments. For all experimental conditions, cells were maintained in media containing 10% charcoal/dextran stripped FBS (substituted for regular FBS) for 48 h to eliminate all endogenous sources of hormones from the FBS. After 48 h, cells were treated with 10 nM E_2_ or an equivalent volume of media containing 0.001% ethanol (vehicle) for 2 or 16 h before lysis.

### 4.3. Molecular Biology Assays

#### 4.3.1. RNA Isolation and cDNA Synthesis

Total RNA was isolated from IVB cells and PVN tissue microdissections using the Zymogen DirectZol kit (cat. #R2051, Zymo Research, Irvine, CA, USA). Total RNA (1.0 mg) was reversed transcribed using the Norgen miRNA cDNA Synthesis kit (cat. #54410, Thorold, ON, Canada) or Invitrogen Superscript IV mRNA synthesis kit for mRNA cDNA (cat. #18091050, Waltham, MA, USA), according to manufacturer instructions.

#### 4.3.2. Reverse Transcription Quantitative PCR (RT-qPCR)

All mRNA transcripts (i.e., miRNA biogenesis components and targets) were quantified by RT-qPCR using the procedures and primers previously described [9]. Each individual biological sample was assayed as triplicate replicates within an assay. We used a standard curve for a small nuclear RNA (SNO87) generated from an expression vector plasmid containing the full-length insert for SNO87. The exact transcript number was calculated by converting 1.0 μg of plasmid (approximately 9.1 × 10^11^ copies) ÷ plasmid size in kilobases. The plasmid was then diluted to 5 ng (4.5 × 10^9^ copies) in a series of 8 dilutions. All standard curves were loaded on the same RT-qPCR plate as experimental samples. The standard curve was generated using QuantStudio software, and each replicate was quantitated against the known SNO87 RNA transcripts. For relative quantification, we used 18S rRNA as a housekeeping gene that has previously been verified as unaffected by E_2_ treatment [52]. Transcripts were quantified relative to vehicle-treated control using the ΔΔCt method [53]. The following conditions were used for the thermocycler: (1) 95 °C for 10 min, (2) 95 °C for 15 s, (3) 59 °C for 20 s, and (4) 72 °C for 12 s in addition to melting curve analysis.

#### 4.3.3. RNA Immunoprecipitation (RIP)

RIP experiments were performed using IVB cells or PVN lysate from OVX animals, as described previously [54]. Briefly, hypothalamic-derived neuronal IVB cells were grown in media containing 10% charcoal-stripped FBS for 48 h, treated with E_2_ (10 nM) or vehicle (0.001% EtOH) for 16 h, washed with 10 mL ice-cold PBS, and then centrifuged at 1500 RPM for 5 min. An equal volume of RNA Lysis Buffer was used to resuspend pellet, then incubated on ice for 5 min to lyse the cells. Magnetic beads were washed and pre-incubated with 2.5 μg AGO1-4 and 2.5 μg rabbit IgG (Wako, Osaka, Japan, Cat. #015-22411; Wako, Cat. #014-22023; Wako, Cat. #016-25501; Abcam, Tokyo, Japan, Cat. #ab85077; EMD Millipore, Burlington, MA, USA, Cat. #PP64B) (negative control). Antibody-coated beads were resuspended and then incubated with cell lysates rotating overnight at 4 °C. Supernatant was removed and beads were washed 5× with RIP wash buffer followed by proteinase K treatment at 55 °C for 30 min.; supernatant was removed from magnetic beads and placed in a new tube for RNA isolation and cDNA synthesis. Input RNA quantity was assessed on the Nanodrop spectrophotometer and analyzed for quality by visualization of the RNA on 2.0% agarose gel. RIP analysis used the ΔΔCt method to determine enrichment fold change relative to IgG controls as previously described [55].

#### 4.3.4. Nuclear RNA Immunoprecipitation (nRIP)

RIP experiments were performed using IVB cells. Briefly, hypothalamic-derived neuronal IVB cells were grown in media containing 10% charcoal-stripped FBS for 48 h, treated with E_2_ (10 nM) or vehicle (0.001% EtOH) for 16 h, washed with 10 mL ice-cold PBS, and then centrifuged at 1500 RPM for 5 min. A total of 500 µL of Nuclear Lysis Buffer (1.28 M sucrose, 40 mM Tris-HCL, 20 mM MgCl_2_, and 4% Triton-X) was used to resuspend pellet, then incubated on ice for 15 min. with vortexing every 5 min. to lyse the cells. Lysate was added to a 1.0 mL Dounce homogenizer and homogenized on ice. Nuclei were then pelleted at 500× *g* for 1 min. The nuclear pellet was resuspended in RIP lysis buffer. Magnetic beads were washed, blocked for 1.0 h. with salmon sperm, and pre-incubated with 2.5 μg AGO1-3 and 2.5 μg rabbit IgG (Wako, Cat. #015-22411; Wako, Cat. #014-22023; Wako, Cat. #016-25501; EMD Millipore, Cat. #PP64B) (negative control). Antibody-coated beads were resuspended and then incubated with cell lysates rotating overnight at 4 °C. Supernatant was removed and beads were washed 5× with RIP wash buffer followed by proteinase K treatment at 55 °C for 30 min.; supernatant was removed from magnetic beads and placed in a new tube for RNA isolation and cDNA synthesis. Input RNA quantity was assessed on the Nanodrop spectrophotometer and analyzed for quality by visualization of the RNA on 2.0% agarose gel. RIP analysis determined enrichment fold change relative to IgG controls as previously described [54].

#### 4.3.5. siRNA Knockdown of hnRNP A1

We reduced the endogenous expression of hnRNP A1 in hypothalamic-derived (IVB) cells using lipid-mediated transient transfection (Fugene LLC, Middleton, WI, USA, cat. # SI-1000) combined with 250 µM specific siRNA (ThermoFisher cat. #50199) or scrambled siRNA as a control (Integrated DNA Technologies, custom-designed sequence). Cells were grown to 60–70% confluence before seeding a 6-well plate at 125,000 cells/well. Seeded cells were transfected 24 h later with FUGENE SI:hnRNP A1 siRNA (3.0 µL:100× µM) and allowed to grow in normal growth media (10% FBS) for 48 h. Transfections were conducted in replicates of six wells across two plates to yield six technical replicates. After 48 h, cells across six wells were collected and split; 2/3 of total collected cell lysate was used for protein extraction for Western blot and the remaining 1/3 used for RNA isolation used for cDNA synthesis for qPCR. Each transfection was repeated for six independent experiments.

### 4.4. Protein Assays

#### 4.4.1. Western Blot

Total protein was isolated from IVB cells and brain tissue samples using a 0.5% NP40 buffer with protease and phosphatase inhibitors (cat. #PI88669, ThermoFisher Scientific, Waltham, MA, USA). Following lysis procedures, protein concentration was determined using a bicinchoninic acid (BCA) assay according to manufacturer instructions (ThermoFisher Scientific, cat. #23225). Total protein (25 μg) was boiled with 4x Laemmli buffer (BioRad, Hercules, CA, USA, cat. #161-0747) at 95 °C for 5 min. before electrophoresis on a 10% polyacrylamide gel. Following gel electrophoresis at 120 V for 1 h, proteins were transferred to Immobilon PVDF membranes (Millipore, cat. #IVPH00010) at 100 V for 1 h at 4 °C. Membranes were blocked with a 1:1 solution of TBS and Odyssey blocking solution (Li-cor Biosciences, cat. #927-50003) for 1 h. at room temperature. Following the blocking procedure, membranes were incubated with primary antibody to detect hnRNP A1 (ThermoFisher Scientific, cat. #XG3640396) overnight with constant agitation at 4 °C. Membranes were then incubated with 1:1000 secondary antibodies (Li-Cor Biosciences, Lincoln, NE, USA) for 1 h. at room temperature. Protein bands were visualized using the Sapphire Azure Biosystems imaging system (Dublin, CA, USA). Densitometry values were calculated after subtracting background and relative fold changes were made compared to vehicle controls.

#### 4.4.2. Immunofluorescence

Hypothalamic PVN-derived neuronal cells (IVB cells) were grown in culture to 80% confluency, washed in PBS, and fixed in 4% paraformaldehyde at room temperate for 10 min. Cells were then permeabilized in 0.5% Triton-X in tris-buffered saline (TBS-T) for 5 min. and washed in TBS 3× 5 min. on a 2D rotator. Cells were blocked for 1 h at room temperature in 5% normal goat serum in 0.5% TBS-T, followed by incubation with primary rabbit monoclonal Argonaute 1 (Cell Signaling, Danvers, MA, USA, cat. #9388S), Argonaute 2 (Cell Signaling, cat. #2897S), Argonaute 3 (Cell Signaling, cat. #5054S), or Argonaute 4 (Cell Signaling, cat. #6913S) antibodies 1:500 in blocking buffer overnight at 4 °C. Cells were then washed 3 × 5 min. at room temperature in TBS. Secondary goat anti-rabbit Alexa Fluor 488 (ThermoFisher Scientific, cat. #A11008) was applied for 1 h at room temperate, followed by 4′,6-diamidino-2-phenylindole (DAPI) nuclear counterstain and 3 × 5 min. TBS washes. Images were captured on the ZOE™ Fluorescent Cell Imager (Bio-Rad, Hercules, CA, USA).

### 4.5. Statistics

Statistical analyses were performed using Prism software (v9.1.0, San Diego, CA, USA). Data were analyzed by two-factor ANOVA followed by Tukey post hoc test for multiple pair-wise comparisons unless otherwise noted in figure captions. Data are displayed as mean ± SEM, and statistical significance was determined at *p <*  0.05.

## 5. Conclusions

The main goal of these studies was to determine the molecular mechanisms underlying the differential expression of E_2_-regulated miRNAs in the hypothalamus of aged female rats. We demonstrated that E_2_ treatment affects miRNA subcellular localization and its association with argonaute proteins differently dependent on the length of time following E_2_ deprivation (i.e., ovariectomy). We also provide strong evidence that the RNA binding protein, hnRNP A1, regulates the transcription of pri-miR-9 and likely plays a role in mature miR-9-5p turnover. Taken together, these data have important implications for considering the optimal timing for hormone replacement therapy, which might be less dependent on age and more related to how long treatment is delayed following surgical menopause.

## Data Availability

All data are contained within the article.
